# Elements of the Practice of Physic, Presenting a View of the Present State of Special Pathology and Therapeutics

**Published:** 1837-04

**Authors:** 


### Art. XI.

1. *Elements of the Practice of Physic, presenting a View of the present State of Special Pathology and Therapeutics.* By David Craigie, m.d. f.r.s.e., Physician to the Royal Infirmary, Edinburgh, &c. Vol. I.—*Edinburgh*, 1836. 8vo. pp. 952.

2. *Elements of the Practice of Medicine.* By Richard Bright, m.d., and Thomas Addison, m.d., Lecturers on the Practice of Medicine at Guy's Hospital. Part I.—*London*, 1836. 8vo. pp. 129.

3. *Elements of Medicine.* Vol. I. *On Morbid Poisons.* By Robert Williams, m.d., Senior Physician to St. Thomas's Hospital. —*London*, 1836. 8vo. pp. 342.

The simultaneous appearance of the three works of which we have transcribed the titles, sufficiently demonstrates the prevalent opinions of the want of an elementary work on practical medicine; as, no doubt, all the authors must have believed that they perceived the vacuum which they purposed to supply by their respective productions. Properly speaking, indeed, the work of Dr. Williams can hardly be regarded as an elementary treatise for the use of students and junior practitioners; as the portion of it already published, at least, is much more calculated to convey the author's own and rather peculiar doctrines respecting the nature and treatment of certain diseases, than to present such general views as are calculated for the instruction and guidance of those who are studying the principles of their profession. For this reason, we shall take another opportunity of reviewing Dr. Williams's book, as it probably will demand a more strict examination than works not professing to be original can be entitled to.

The two other works noticed above, although composed with the same view of teaching practical medicine to the uninstructed, are extremely different in their character: in most particulars, indeed, they may be regarded as the opposites of each other. And yet they are both excellent in their respective ways; and we believe that, if read together, the student would derive much more advantage than from confining his attention to either. Dr. Craigie's work is elaborate and learned, full to overflowing of matter, comprehending almost every thing, whether in the way of fact or opinion, of theory or practice, from the days of Hippocrates to the present time; while that of Drs. Bright and Addison presents the reader with a simple outline or sketch of what they consider to be the soundest views and doctrines of the schools as at present known and taught. When we say that we think the student would do well to take both for his guide in study, we mean that, by reading the Elements of Drs. Bright and Addison in the first place, he might obtain a clear conception of the principal facts both of pathology and practice which it is important to have impressed on the mind, while Dr. Craigie's Treatise would supply him with ample materials for exercising his reasoning and his judgment on every point which might appear to him doubtful or unsatisfactory in the brief statements of the other. For a mere beginner, indeed, Dr. Craigie's book is too vast and comprehensive; it would infallibly puzzle and perplex him by the number and variety of objects presented to his mind on almost every subject treated of: but to the student, when somewhat advanced, who wished to make himself master of what is known on any particular disease or class of diseases, or to the junior practitioner, who is anxious to prosecute that best of all modes of study, namely the study of diseases as they appear in nature, in conjunction with their history, pathology, and treatment, as detailed in books, it must prove of great value. It is, in fact, better calculated to serve as a work of reference than as a manual for the student. Its very comprehensiveness, complexity, and learning prevent the pictures of disease which it contains from making that strong and definite impression on the mind which works of infinitely less merit are calculated to do. Indeed, it is with such works as the *Cyclopædia and Dictionary of Practical Medicine*, that Dr. Craigie's work should be compared; it resembles the latter more particularly in its great learning, and in that misty perplexity left in the mind by its perusal, a necessary consequence of an attempt to state briefly and consecutively a vast variety of views and opinions on any particular subject. It has, alas, but few of those slight but striking sketches drawn from nature, the graphic distinctness and boldness of which bespeak their source, and which leave a greater impression on the mind of the reader than all the masses of ideas that labour and learning can pile together.

The small work of Drs. Bright and Addison is, on the other hand, truly calculated, as it is intended, for the student. It will materially aid his progress while attending the lectures of his teachers, or reflecting on the cases he has seen in the hospital; it may even form a safe and useful guide in practice to the uninquiring and routine practitioner; but it is vastly too meager to satisfy the wants of any one who is really desirous of knowing his profession, or to be of much use in allaying or removing the doubts and difficulties which so much beset the path of the young practitioner, and probably most so the path of the best informed and most scientific.

In going over the treatment of the different forms of fever (intermittent, remittent, and continued,) treated of in this first fasciculus, we marked several passages for comment, which our limited space prevents our noticing at length. We must, however, express our opinion that the authors are too fond of the employment of active mercurials and purgatives in fever generally; and that, in those varieties, whether continued or remittent, which are complicated with morbid irritation or inflammation of the mucous membrane of the intestines, they recommend a practice often the reverse of what our experience leads us to regard as the best.

Thus, at p. 60, in treating of remittent fever attended with purging, we are told to give calomel and rhubarb, hydrargyrum cum cretâ, castor oil, chalk, opium, &c.; but leeching the abdomen or anus, often the most effectual remedy, is never mentioned. Again, in the treatment of the infantile remittent fever, (p. 69-70,) we think the practice recommended is, generally speaking, much too irritating and drastic. Calomel and purgatives seem to be regarded as the sole means of quieting the gastric and intestinal irritation; leeching is never spoken of; and the warm bath, so peculiarly soothing in such cases, is slurred over with the incidental remark, that "the little patients will often derive considerable benefit from its occasional use." We are the more surprised at this *medicina perturbatrix* in such cases, as, in the subsequent page (71), we observe some excellent cautions against adding to the irritation of the digestive organs by mercurials. We think the article on the infantile remittent fever is especially defective in not sufficiently discriminating the chronic from the acute, the incipient from the strongly marked form of the disease. The same disposition to recommend mercurials and active purging in excess is conspicuous in the treatment of continued fever. "One or two evacuations should be procured daily, and, as they are for the most part unnatural in appearance, we generally prefer a mercurial purge to any other. Three, four, or five grains of calomel, with ten or twelve of rhubarb or of jalap, answer very well; or a saline purge with manna, or compound infusion of senna, may now and then be substituted." (P. 100.) We hope we have given sufficient proof, in the present number, that we are no abettors or sectators of the Broussaian doctrine of fever; but, on the plain principles of rational pathology, confirmed by experience, we protest against this old-fashioned and empirical method of treating fever.

Dr. Craigie's volume, which comprehends the whole diseases included in the classes *Fevers*, *Cutaneous Inflammations*, and *Mucous Inflammations*, deters criticism by its very extent; although, did our limits permit, we could point out many statements and opinions which are not in accordance with our views. On the whole, however, we are disposed to admit that, in the execution of his work, he has not fallen much short of the lofty aim he had in view in composing it.

"It has been my study throughout to present such a view of special pathology and therapeutics as may be justly said to be the united result of the observation and researches of all the ablest pathologists and physicians, by whom the science of medicine has been cultivated, and by whom its art has been simplified, improved, and rendered energetic. In the prosecution of this method, I have applied to medicine the same principles which have been long applied to the physical and physiological sciences in general, and to morals, and to law, public and particular. The facts and classes of facts recorded by different observers, I have examined, compared, and analyzed; where the results have varied, I have studied to discover the causes on which the discordance depended; where they have been proved to be erroneous, I have studied to rectify them by the result of the labours of others, or by my own experience; when they have been defective, I have by the same means endeavoured to supply what is wanting; and, from the whole, I have cautiously deduced correct and legitimate general principles; and have specified the points in which particular exceptions become requisite." (*Pref.* vii.)

As a specimen of the work, we extract the short account which he gives of a disease with which we have been all so recently familiar—*Influenza.*

"In certain seasons, however, catarrh prevails most extensively, and betrays, by the number of persons whom it attacks, a character decidedly epidemic. This variety of the disorder, which is called influenza, differs from the ordinary catarrh, chiefly in the greater suddenness of its attack, in the symptoms of fever and muscular languor and weakness being in general more considerable, in those of the tracheo-bronchial membrane being more severe, in the mucous membrane of the alimentary canal being often affected at the same time, and in some instances in its being associated with a decided rheumatic tendency. This variety of catarrh has been believed by many to be infectious, or capable of being propagated from one individual to another, and the disease was very generally denominated contagious catarrh (*catarrhus contagiosus.*) This opinion, however, must have been adopted on very inadequate grounds, and without sufficient attention to the manner in which epidemic catarrh appears, prevails, and declines. The following circumstances show, I conceive, that the complaint, whatever be its origin and character, is not one of contagious properties.

"1. Catarrh, when epidemic or prevailing extensively, is preceded by a peculiar train of atmospheric phenomena, and by winds in general blowing in a particular direction.

"2. Catarrh when epidemic appears very suddenly, and affects at one and the same time great numbers of individuals, sometimes thousands, in the course of a few days or weeks. In the spring of 1832 and 1833, it appeared in this manner, and affected at once great numbers of individuals, who could have had no communication, both in London and Edinburgh.

"3. Catarrh, when epidemic, passes rapidly through a community, and cases completely and entirely for that season. The catarrhal epidemics of the seasons now mentioned were completed in Edinburgh in the course of about five weeks.

"4. A system of separation of the sound from the sickly seems to be quite ineffectual in the case of influenza.

"While, in short, there are no reasons to believe catarrh infectious, many concur to show that it is epidemic, or dependent upon some constitution of the atmosphere and weather. The irritative cause, which produces epidemic catarrh, appears to be one which operates much more extensively than that of sporadic catarrh. While the latter acts chiefly if not exclusively on the rhino-tracheo-bronchial mucous membrane, the influence of the former is seen not only on that but in the gastro-enteric mucous membrane, and, I think, so far as I have observed, on the skin and the fibrous tissues and aponeurotic sheaths. Influenza is not always a tracheo-bronchial disorder. It is often one which affects the system generally, and the vascular system of all the membranes; and this I regard as the chief cause of the more intense degree of feebleness and lassitude with which it is almost invariably accompanied, and of the long tract of weakness and delicate health by which it is often followed. The tendency of epidemic catarrh is quite similar to that of sporadic catarrh. But it is in those who have already laboured under catarrh, or in whom the bronchial membrane is weakened by repeated attacks, and therefore in the aged, very liable to induce the worst and most unmanageable forms of bronchial inflammation, that named spurious or bastard peripneumony." (P. 823.)

